# Targeting Activated Hepatic Stellate Cells Using Collagen-Binding Chitosan Nanoparticles for siRNA Delivery to Fibrotic Livers

**DOI:** 10.3390/pharmaceutics12060590

**Published:** 2020-06-25

**Authors:** Menna Azzam, Sara El Safy, Sarah A. Abdelgelil, Ralf Weiskirchen, Anastasia Asimakopoulou, Federica de Lorenzi, Twan Lammers, Samar Mansour, Salma Tammam

**Affiliations:** 1 Department of Pharmaceutical Technology, Faculty of Pharmacy & Biotechnology, The German University in Cairo (GUC), 11835 Cairo, Egypt; menna.khaledd@gmail.com (M.A.); saraelsafy@live.com (S.E.S.); s.a.radwan95@gmail.com (S.A.A.); samar.mansour@guc.edu.eg (S.M.); 2 Institute of Molecular Pathobiochemistry, Experimental Gene Therapy and Clinical Chemistry (IFMPEGKC), RWTH University Hospital, D-52074 Aachen, Germany; rweiskirchen@ukaachen.de (R.W.); anast.asimakopoulou@gmail.com (A.A.); 3 Department of Nanomedicine and Theranostics, Institute for Experimental Molecular Imaging, RWTH Aachen University Clinic, D-52074 Aachen, Germany; fdelorenzi@ukaachen.de (F.d.L.); tlammers@ukaachen.de (T.L.)

**Keywords:** liver fibrosis, TGF-β, collagen, chitosan nanoparticles, active targeting, siRNA delivery, activated hepatic stellate cells, collagenase

## Abstract

Activated hepatic stellate cells (aHSCs) are the main orchestrators of the fibrotic cascade in inflamed livers, with transforming growth factor-beta (TGF-β) being the most potent pro-fibrotic cytokine. Hence, aHSCs serve as interesting therapeutic targets. However, drug delivery to aHSCs is hindered by excessive collagen deposition in the extracellular matrix (ECM) and capillarization of liver sinusoids. Chitosan-nanoparticles (CS-NPs) show intrinsic affinity for collagen, holding potential for drug delivery to fibrotic livers. Here, we employed CS-NPs for anti-TGF-β siRNA delivery, promoting delivery into aHSCs via modification with platelet-derived growth factor receptor-beta binding peptides. In-vitro experiments using aHSCs demonstrated the association of unmodified CS-NPs to the collagen-rich ECM, with reduced intracellular accumulation. Peptide-modified CS-NPs showed a higher propensity to localize intracellularly; however, this was only the case upon ECM-collagen reduction via collagenase treatment. Peptide-modified CS-NPs were more potent than unmodified CS-NPs in reducing TGF-β expression, implying that while collagen binding promotes liver accumulation, it hinders cell-specific siRNA delivery. In-vivo, CS-NPs successfully accumulated in fibrotic livers via collagen binding. Similar to in-vitro findings, when mice were pretreated with collagenase-loaded CS-NPs, the accumulation of peptide-modified NPs increased. Our findings demonstrate the usefulness of NPs modification with targeting ligands and collagenase treatment for aHSCs targeting and highlight the importance of chitosan–collagen binding in drug delivery to fibrotic diseases.

## 1. Introduction

Liver fibrosis results from chronic hepatic injury by various insults. During hepatic fibrosis, the continuous accumulation of collagen rich extracellular matrix (ECM) leads to scar deposition. If left untreated, such scarring could convert into cirrhosis and eventually progress to liver failure with an increased risk of the development of hepatocellular carcinoma (HCC) [1,2]. Hepatic stellate cells (HSCs) are the main orchestrators of the fibrotic cascade [3]. HSCs are located between the sinusoidal endothelium and hepatocytes in the space of Disse and, under normal conditions, exist in a quiescent state [1,2]. However, in response to liver injury, they become activated. Activated hepatic stellate cells (aHSCs) show an increased expression of α-smooth muscle actin, enhancing their contractility, which contributes to vascular distortion and increased vascular resistance promoting portal hypertension in fibrotic livers [3]. Moreover, aHSCs secrete almost 80% of total fibrillar collagen I that deposits in the fibrotic liver [3,4,5]. Therefore, this cell type presents an interesting target for the treatment of liver fibrosis [6].

HSC activation is regulated by several mediators in a paracrine and autocrine manner [3,7]. Among them, transforming growth factor beta (TGF-β1) is the most potent cytokine responsible for the regulation of the HSC phenotype and the most potent stimulus for collagen I production [3,7]. In fact, TGF-β1 is believed to maintain the activated cell population by reducing aHSC apoptosis, enhancing aHSC activation and at the same time increasing ECM deposition, as well as by increasing the expression of matrix proteins and reducing the levels of ECM degrading enzymes [7]. It can therefore be concluded that inhibiting the expression of this cytokine would result in a reduction of the activated phenotype and potentially in the reversal of fibrosis. However, given its wide expression and involvement in several cellular processes and cascades [8,9,10], its unspecific inhibition would not be free of side effects. Therefore, a targeted delivery system, capable of reducing TGF-β1 production by the aHSCs in the fibrotic liver, might serve as a potential approach for the treatment of liver fibrosis.

Drug targeting using nanoparticles (NPs) provides a means to improve pharmacological interventions that bear therapeutic potential but suffer from inappropriate biodistribution. The success of pharmacotherapies is often hampered by insufficient supply to the pathological site and/or by accumulation in off-target tissues, resulting in undesirable adverse effects [2,11]. For drug targeting to aHSCs, NPs should initially encapsulate the active molecule without compromising its therapeutic activity. This is of particular importance when sensitive macromolecular compounds, such as siRNA or proteins, are to be delivered [12,13]. Following in-vivo administration, NPs should then localize in the space of the Disse, where the aHSCs reside. Once targeted to the space of Disse, the NPs should show gradual accumulation in target cells. In healthy livers, the liver sinusoidal endothelial cells (LSECs) fenestrae offer a permeation window enabling nanoparticulate matter to localize into the space the Disse [14]. During fibrosis, dramatic changes in the LSEC phenotype are observed, of which “capillarization” constitutes an important barrier hindering NPs’ access to the aHSCs. The loss of fenestrae, accompanied by the abnormal deposition of a basement membrane matrix, limits the access of the NPs to their target cells [14]. Consequently, smaller NPs would have a larger chance of translocating into the liver, in areas where fenestrae are still accessible [2]. Once in the space of Disse, the NPs could then show enhanced accumulation in the aHSCs if they are targeted to one of the surface receptors (over-) expressed by aHSCs. Several receptors are present on the aHSCs surface and ligands have been identified that can be used to improve the delivery of various antifibrotic agents [6]. As a key example, the platelet-derived growth factor receptor beta (PDGFR-β) is highly over-expressed on aHSCs and shows much lower expression levels on other cells in the liver [15,16]. Accordingly, several PDGFR-β-binding peptides have been reported [17,18] and have served as interesting molecular targets through which enhanced NP–aHSC interactions can be achieved.

Chitosan is a biodegradable polymer that has been widely employed in the development of NPs for drug delivery, in particular for protein and nucleic acid delivery. This is mainly attributed to the NPs’ hydrophilicity as well as to the fact that their formulation by ionotropic gelation is devoid of organic solvents and the use of heat [2,12,19,20,21,22,23]. Additionally, and highly important in the context of fibrotic diseases, chitosan has been reported to bind and interact with collagen [24]. We recently demonstrated the ability of small (100 nm) chitosan NPs to accumulate in fibrotic livers following intravenous administration as a function of their collagen binding properties [25].

**Figure 1:**
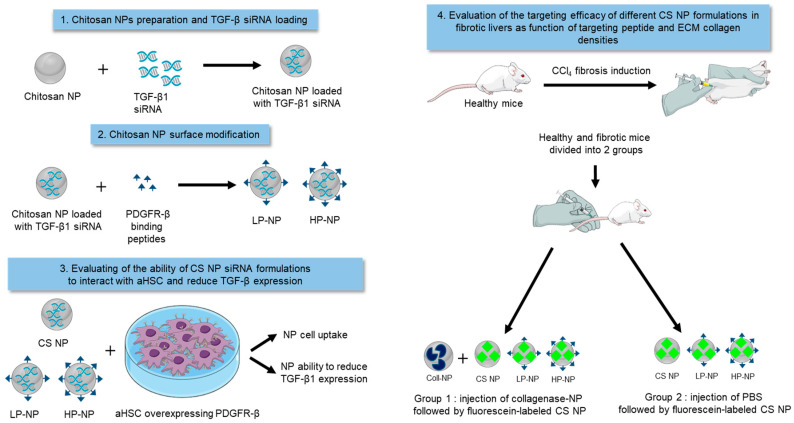
Schematic representation of the experimental workflow.

Taking the above notions into account, we reasoned that the development of small TGF-β1 siRNA chitosan NPs might offer an interesting approach for aHSC deactivation and the treatment of liver fibrosis. Accordingly, in the present study, we developed small TGF-β-siRNA-loaded chitosan NPs and we modified them with different densities of PDGFR-β-binding peptides, given the effect of targeting ligand density on the efficacy of NPs active targeting [2,20]. These resulting NPs were tested for their ability to accumulate in aHSCs and to reduce the expression of TGF-β1 in-vitro, as a function of targeting peptide density and collagen density in the ECM. In addition, the ability of NPs to accumulate in fibrotic livers was assessed in-vivo. To study the effect of ECM collagen content on NP accumulation in-vivo, the liver localization of the developed chitosan NPs was determined when administered on their own and in combination with collagenase-containing NPs (Figure 1).

## 2. Materials and Methods

### 2.1. Nanoparticle Preparation and Characterization

Chitosan NPs (CS-NPs) were formulated by the ionotropic gelation method as detailed in earlier work [20]. Briefly, in a ratio of 1:1, 0.125% (w/v) sodium tripolyphosphate (TPP; Mistral Chemicals UK) was added to 0.4% (w/v) chitosan solution (low molecular weight, Sigma Aldrich, Taufkirchen, Germany) dissolved in 1% acetic acid, pH 4.0 and stirred for 30 min at 1000 rpm. NPs average hydrodynamic diameter (HD) and zeta potential (ZP) were determined using a Malvern Zetasizer Nano ZS90 (Malvern Instruments Ltd., Malvern, UK), from three independently prepared batches of NPs each analyzed in triplicates at 25 °C. The results were expressed as the mean ± standard deviation (SD). For morphological analysis, a drop of the NP suspension was placed on carbon film and stained with uranyl acetate (1% w/v) before investigation by transmission electron microscopy (TEM) (JEOL, Japan). Additionally, CS-NP suspension was spread on glass slides and examined by field-emission scanning electron microscopy (SEM) using a LEO SUPRA 55 microscope (Carl Zeiss, Reutlingen, Germany).

### 2.2. In-Vitro CS-NPs Cytotoxicity Studies

A continuous murine cell line termed GRX with aHSC phenotype [26] and human embryonic kidney cell line HEK293 [27] were selected as the HSC cell line of choice and a control cell line, respectively, following the quantification of PDGFR-β and TGF-β1 basal gene expression levels (Supplementary Materials). Cells were cultured in Dulbecco’s Modified Eagle Medium (DMEM) supplemented with 10% fetal bovine serum (FBS), 1% penicillin/streptomycin, 1% sodium pyruvate and 2 mM L-glutamine at 37 °C in a humidified atmosphere containing 5% CO2. Cells were seeded in 96-well plates at a density of 30,000 cells/well and incubated for 24 h. The cells were then treated with increasing concentrations (0.1, 0.5, 1, 1.5, 2, 3 and 4 mg/mL) of CS-NPs and incubated for an additional 24-h period. Thereafter, the cell viability was determined by the MTT assay essentially as described before [28]. Untreated cells were used as a reference for cell viability determination. The experiment was conducted in triplicates and results were expressed as the mean ± SD, for n = 4.

### 2.3. Nanoparticle Modification with PDGFR-β Binding Peptide

CS-NPs were modified with different densities of the PDGFR-β-binding peptide CIPLPPPSRPFFK [18] (Biomatik, USA) using the short-chain amine-thiol crosslinker succinimidyl 3-(2-pyridyldithio)propionate) (SPDP) (ThermoFisher Scientific, Schwerte, Germany), via a stepwise approach that was optimized in earlier work from our group [20,25]. Briefly, SPDP was dissolved in dimethyl sulfoxide (DMSO ) and added to CS-NPs at a final concentration of 0.9 mM. Successful SPDP tagging to CS-NPs was confirmed using the pyridine-2-thione assay as described before [20,25]. The SPDP-modified NPs were incubated with increasing concentrations of fluorescein labeled CIPLPPPSRPFFK (Biomatik, Wilmington, DE, USA)—with a final concentration of 0.1, 0.15, 0.3 and 0.5 mM peptide—and allowed to react overnight. Following overnight incubation, the NPs were centrifuged at 14,000 rpm at room temperature for 30 min and the extent of peptide tagging on the NPs’ surface was determined by fluorometry at λex 490 nm; λem 525 nm for n = 3. From the results obtained, two concentrations of tagged peptide were selected for further experiments carrying a low (LP-NP) and a high (HP-NP) peptide density on the NPs’ surface. The number of peptides per NP was calculated as detailed in [20] using Equations (1)–(4).

Equation (1): The volume of polymer used in NP formulation = the mass of polymer used in NP formulation/the density of polymer.

Equation (2): The volume of NPs = 4/3π r3, where r is the NP radius.

Equation (3): The number of NPs = the volume of polymer used in NP formulation/the volume of one NP. 

Equation (4): The number of peptides per NP = (concentration of peptide tagged (M) * Avogadro’s number)/the number of NPs.

### 2.4. Nanoparticle Loading and Determination of Encapsulation Efficiency

A fluorescein-labeled model oligonucleotide (MO) (TCA CAA TTG CCA GTT AAC GTC T, Bio Basic Inc., Canada) was initially used to assess the ability of CS-NPs in encapsulating nucleic acid therapeutics and for ease of quantification. MO was initially added to the chitosan solution followed by the addition of TPP, as detailed above. MO was added in two final concentrations (0.05 and 0.15 μM). In both cases, CS-NPs were centrifuged at 14,000 rpm for 30 min at room temperature to remove free MO, while the encapsulated concentration of MO and encapsulation efficiency (EE%) were determined by fluorometry at λex 490 nm; λem 525 nm. EE% was calculated as follows: EE% = [encapsulated concentration of MO in NP/total concentration of MO] × 100% and the results were expressed as %w/w, for n = 3.

### 2.5. In-Vitro CS-NPs Association in GRX and HEK293 Cells

To determine the effect of peptide density on the extent of NP association in the immortalized hepatic stellate cell line GRX and HEK293 cells, NPs were loaded with MO and modified with a low and high density of peptide as detailed earlier. GRX and HEK293 cells were seeded at a density of 30,000 cells/well. MO-loaded CS-NPs, LP-NPs and HP-NPs were added to the cells at a concentration of 0.5, 1, 1.5 and 2 mg/mL. NP formulations were incubated for 24 h. Thereafter, the cells were washed with phosphate-buffered saline (PBS) and the extent of NP association was determined by fluorometry λex: 490nm and λem: 520 using NP curves. The experiment was conducted in triplicates and the results were expressed as the mean ± SD. In order to evaluate the role of collagen in NP association, the experiment was repeated using cells pre-incubated with 0.2 mg/mL collagenase for 1 h. The collagenase concentration used was predetermined based on a collagenase cell cytotoxicity study (Supplementary Materials). After 1 h, the collagenase-containing cell culture media were aspirated and MO-loaded CS-NPs, LP-NPs and HP-NPs were added to the cells at a concentration of 1.5 mg/mL and incubated for 24 h. The concentration of NPs associated with the cells was determined by fluorometry using the NP calibration curves. The experiment was conducted in triplicates and the results were expressed as the mean ± SD. GRX and HEK293 cells were seeded on cover slips in 24-well plates at a density of 110,000 cell per well. After 24 h, MO-loaded CS-NPs were added to the cells and incubated for 24 h. After 24 h, NPs containing media was aspirated, and cells were washed with PBS. Nuclei were fixed, stained with 4′,6-diamidino-2-phenylindole (DAPI) and imaged under fluorescence in a Nikon eclipse 80i microscope (Tokyo, Japan).

### 2.6. Quantitative Real-Time PCR Analysis

Four TGF-β1 siRNA sequences were purchased from Qiagen (Hilden, Germany), (Table S3). TGF-β1 siRNA were encapsulated into CS-NPs in a similar manner to MO, also using a 0.15 μM final concentration of siRNA per preparation. TGF-β1 siRNA-loaded CS-NPs were either used as such in their unmodified form, or modified with a low and high density of PDGFR-β as detailed earlier. GRX cells were seeded at a density of 400,000 cells/well and treated with the four siRNA targeting TGF-β1 in five different forms: (i) in naked form without transfection reagent, (ii) pre-complexed with Lipofectamine 2000 (ThermoFisher Scientific), (iii) encapsulated in unmodified NPs, (iv) in LP-NPs, and (v) in HP-NPs. In all cases, the cells were treated with a final concentration of 1 µM per well in serum-free Opti-MEM (Gibco™, ThermoFisher Scientific) and left to incubate for 24 h at 37 °C in a humidified atmosphere containing 5% CO_2_. Untreated cells were used as controls. After incubation, the cells were harvested and RNA extracted for RT-qPCR (as detailed in Supplementary Materials). The values of target genes were normalized to the housekeeping gene GAPDH, using the 2-ΔΔCT method and results were represented as mean relative TGF-β mRNA expression ± SD for n = 3. The same experiment was repeated for siRNA sequences 1 and 4 in an identical manner. However, in this cell culture experiment, the culture media was supplemented with 10% FBS.

### 2.7. In-Vivo Nanoparticle Biodistribution

Male Swiss albino mice (8 weeks old, weighing 25–30 g), were purchased from Theodor Bilharz Research Institute (TBRI) Cairo, Egypt. The mice were initially divided into two groups (healthy controls and fibrotic mice). Chronic liver damage was induced by intra-peritoneal (IP) injection of 10% CCl_4_ in olive oil (2.5 µL/g body weight) twice a week for one month [29]. The second group served as the healthy controls and received the same volume of olive oil through IP injections. To confirm the establishment of fibrosis after 4 weeks of CCl_4_ injections, one mouse was sacrificed by cervical dislocation and liver tissue samples were obtained, for histopathological investigations. Briefly, livers were washed in tap water and then in serial dilutions of methyl, ethyl and absolute ethyl alcohol to achieve dehydration of the tissue. Specimens were cleared in xylene and embedded in paraffin at 56 °C in a hot air oven for 24 h. Paraffin tissue blocks were prepared for sectioning at 4-μm thicknesses by sledge microtome. The obtained tissue sections were collected on glass slides, de-paraffinized, stained with hematoxylin and eosin (H&E) for examination under the light microscope [30]. To determine whether chitosan collagen binding affects the NP in-vivo biodistribution and whether the excess deposition of collagen in fibrotic livers would facilitate NP accumulation in fibrotic livers Collagenase-loaded chitosan nanoparticles were prepared as detailed in earlier work from our group [25]. The healthy and fibrotic groups were divided each into two groups; the first group received collagenase-NPs intravenously (IV) for one week followed by fluorescent CS-NPs, LP-NPs and HP-NPs, while the second group received PBS instead of collagenase-NPs followed by fluorescent CS-NPs, LP-NPs and HP-NPs. A total of 0.8 mg NPs per mouse was administered intravenously through the tail vein. The total NPs amount was divided over 3 doses given at 2-h intervals [2]. One hour after the last dose, the mice were sacrificed by cervical dislocation and liver tissue samples were harvested. Livers were homogenized in PBS to yield a homogenate with a final concentration of 0.25 g/mL [2]. The concentration of NPs in the livers was then determined by fluorometry using CS-NPs, LP-NPs and HP-NPs calibration curves constructed in liver homogenates. Additionally, to determine whether CS-NPs distribution to other organs changed as a function of fibrosis and excessive collagen deposition, healthy and fibrotic animals were divided into two groups. The first group received intravenous fluorescent CS-NPs (0.8 mg over 3 doses), whereas the second group received the same volume of PBS. One hour after the last dose, the mice were sacrificed by cervical dislocation, and livers, spleens, kidneys, brains and lungs were harvested and homogenized in PBS to yield a homogenate with a final tissue concentration of 0.25 g/mL. The fluorescent intensities of organ homogenates obtained from CS-NP-receiving mice were normalized to fluorescent intensities of organ homogenates form PBS-receiving mice. Figure S1 provides a schematic representation of the in-vivo study Animal care and all experimental procedures were conducted according to the ethical guidelines of the Research Ethics Committee of Faculty of Pharmacy, German University in Cairo (GUC), Project ID: 2019-03-TC-SMH-MK (approved on 16 February 2019).

### 2.8. Statistical Analysis

Statistical analysis was performed by GraphPad InStat software (GraphPad Software, La Jolla, CA, USA) using one-way analysis of variance test (ANOVA). *p*-values <0.05 were considered statistically significant.

## 3. Results and Discussion

### 3.1. Nanoparticle Synthesis and Characterization

**Figure 2:**
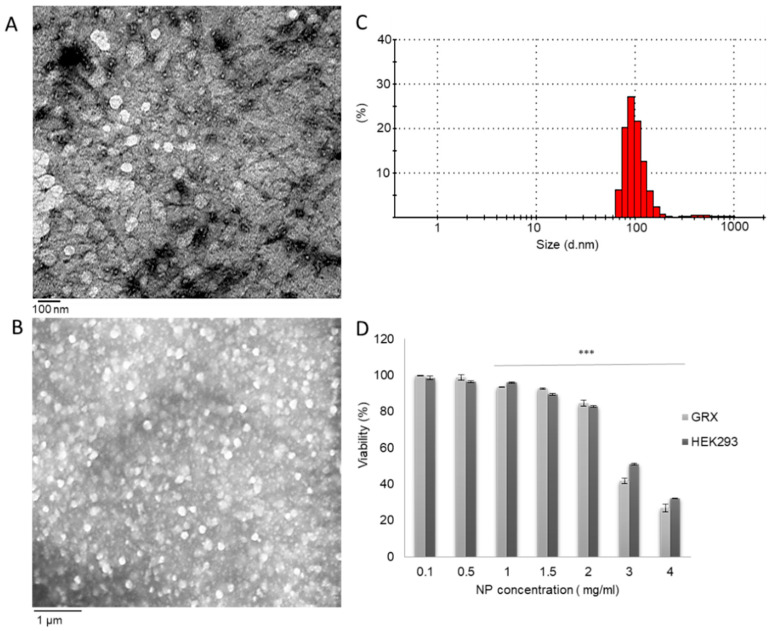
(**A**) Transmission electron microscopy and (**B**) scanning electron microscopy images obtained for chitosan nanoparticles (**C**) Hydrodynamic diameter distribution characterization by Dynamic Light Scattering. (**D**) The effect of chitosan nanoparticles (CS-NPs) on GRX and HEK293 cell viability, as determined by the MTT assay. The results are presented as the mean ± SD viability relative to untreated cells (n = 4). Statistical analysis was performed by GraphPad InStat software using a one-way analysis of variance test (ANOVA), where (***) *p* < 0.001.

In the case of NP delivery to the space of Disse in fibrotic livers, excessive collagen deposition may act both as a hurdle and as a mediator. The capillarization resulting from fibrosis reduces NPs entry from the circulation into the space of Disse. At the same time, however, if the NPs have the ability to interact with and bind to collagen, this would potentially lead to enhanced in-vivo accumulation. In such cases, the NPs need to evade sequestration by the reticuloendothelial system (RES) and they should therefore be formulated from relatively hydrophilic materials and possess relatively small hydrodynamic diameters [31]. Chitosan is a hydrophilic polymer that has been demonstrated to be able to interact with collagen [24]. We recently demonstrated that CS-NPs have the ability to bind to collagen with relatively high affinity, making the use of collagen targeting peptides unnecessary [25]. Here, CS-NPs had an average hydrodynamic diameter of 110 ± 6 nm and a zeta potential (ZP) of 35 ± 1 mV. TEM (Figure 2A) and SEM (Figure 2B) analysis showed that the NPs appeared spherical in shape and in an unaggregated state with a uniform particle size distribution, as indicated by the size distribution charts obtained using Zetasizer (Figure 2C). In addition to the evasion of the RES, this small size can increase the NP’s ability to access the space of Disse via the remaining fenestrae [32].

While utilizing the intrinsic ability of CS-NPs to bind to collagen is an interesting approach to increase NP concentrations in fibrotic livers, these NPs may suffer from collagen sequestration when it comes to interaction with their target cells. However, if such NPs hold the potential to bind to collagen and at the same time interact specifically with target cells, a synergistic targeting benefit could potentially be achieved. Therefore, to enhance their interaction with the aHSCs, CS-NPs were modified with different densities of PDGFR-β-binding peptides. PDGFR-β is abundantly expressed on the cell surface of aHSCs and could serve as a specific means for targeting [33]. In this work, IPLPPPSRPFFK [18] was selected as the targeting peptide. It is obvious that, in addition to the correct choice of targeting ligand, the success of active targeting also depends on ligand orientation and ligand density [13,20,21,25]. Therefore, a stepwise peptide tagging approach, optimized in earlier work [20,25], was adopted. To this end, a cysteine (Cys) residue was initially added to the N-terminus of the targeting peptide. The thiol groups of the inserted Cys moieties enable linking to the amine group in the NPs, via the use of SPDP as an amine-thiol crosslinker. The presence of amine groups on the NPs surface is evident from the overall positive ZP observed for CS-NPs. Given that only one thiol group is present in the targeting peptide, controlling peptide orientation is a function of the cross-linker used. For this reason, CS-NPs were initially allowed to react with SPDP, forming a thiol-reactive intermediate whose formation was detected quantitatively by the pryridne-2-thione assay [20,25,34]. We recently demonstrated that the density of SPDP on the surface of the NPs is not a contributing factor to the density of peptide tagged [25]. Hence, we here only used one SPDP concentration (0.9 mM) to obtain SPDP-NPs with an SPDP concentration corresponding to 42.2 ± 1.4 µM. The thiol-reactive NP intermediates were then reacted with increasing concentrations of the thiol-bearing fluorescent targeting peptide. As the concentration of peptide added to SPDP-NPs increased, the concentration of peptide tagged also increased, until a plateau was achieved, indicating NP surface saturation (Figure S2). At saturation, the peptide density on the NP surface was termed high-peptide density (HP; corresponding to ~2250 peptides per NP) and accordingly a low-peptide density (LP; corresponding to ~892 peptides per NP) was selected

### 3.2. In-Vitro Association of Chitosan Nanoparticles by HEK293 and GRX Cells

To evaluate the ability of NPs to interact with aHSCs as a function of collagen density in the ECM and targeting peptide density on the surface of the NPs, two cell lines were used, GRX and HEK293 cells. GRX cells are a continuous murine cell line with an aHSCs phenotype [26] and the ability to secrete collagen in-vitro [35]. These cells were selected given the higher expression levels of PDGFR-β and TGF-β1 in GRX cells and their much lower expression in the control cell line HEK293 cells (Figure S3). Figure 2D shows the viability obtained when the cells were treated with increasing concentrations of CS-NPs. In this set of experiments, both GRX and HEK293 cells showed minimal loss in viability at NP concentrations up to 2 mg/mL. The IC50 value was 2.5 mg/mL for GRX cells and 2.8 mg/mL for HEK293 cells. All subsequent experiments were consequently conducted at NP concentrations that were below 2 mg/mL.

**Figure 3:**
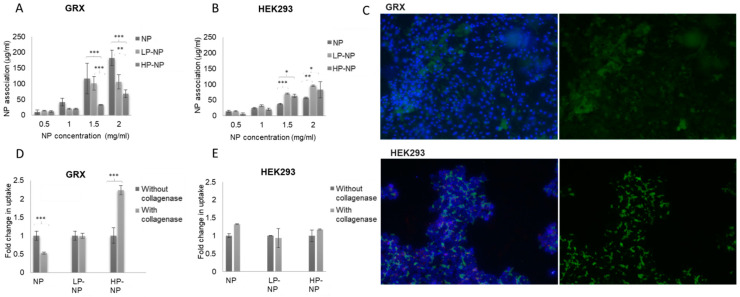
CS-NP association in GRX and in HEK293 cells as a function of NP peptide density and extracellular matrix (ECM )collagen density. CS-NP association in (**A**) GRX and (**B**) HEK293 cells results expressed as the mean ± SD concentration of NPs associated by the cells (n = 3). (**C**) Fluorescent microscopy studies of model oligo (MO)-loaded CS-NPs; when incubated for 24 h with GRX and HEK 293 cells, nuclei appear blue due to DAPI staining, whereas CS-NPs appear green. CS-NP association following pretreatment with collagenase in (**D**) GRX and (**E**) HEK293 cells; the results are expressed as the mean ± SD fold change in association as result of collagenase pre-treatment (n = 3). Statistical analysis was performed by GraphPad InStat software using a one-way analysis of variance test (ANOVA), where (*) *p* < 0.05, (**) *p* < 0.01 and (***) *p* < 0.001.

CS-NPs were loaded with a fluorescent model oligonucleotide (MO), to enable the quantification of NPs association. An encapsulation efficiency of 84.8 ± 5.9% w/w and 101.8 ± 0.2% w/w were observed when MO was added to CS-NPs at a final concentration of 0.05 and 0.15 μM, respectively. While NP loading did not affect NP HD, a reduction in NPs ZP was observed (Figure S4), which might indicate that a portion of MO is available at the particle surface. When both cell lines were incubated with increasing concentrations of MO-containing NPs for 24 h, a concentration-dependent association was observed (Figure 3A,B). Surprisingly, in GRX cells, despite higher expression levels of PDGFR-β, unmodified CS-NPs showed higher association than peptide-modified NPs (for both LP-NPs or HP-NPs). Somewhat surprisingly, the highest peptide density even resulted in the lowest association. NP association refers to the association of the NPs with the cell membrane, the produced ECM and/or their intracellular localization [36]. Given the ability of chitosan to associate with collagen [24,25] and given the high collagen content of the ECM of GRX cells [35], it seems obvious that high association observed for CS-NPs results from NPs binding to the collagen-rich ECM. The latter is underpinned by the notion that peptide modification resulted in reduced NP association in a peptide density-dependent manner, given the ability of the peptide to shield the collagen-binding capacity of chitosan via steric hindrance. This is also obvious from the fluorescent microscopy images obtained when GRX and HEK293 cells were treated with MO-loaded CS-NPs (Figure 3C). In HEK293 cells, NPs appear to localize intracellularly in the cytoplasm, as opposed to the more diffuse appearance observed when GRX cells where incubated, showing hardly any green fluorescence within the cells. When the association experiment was repeated following cell pretreatment with collagenase, a different trend was found (Figure 3D,E). Since collagenase is a collagen-degrading enzyme [37] and potently reduces the collagen density in the ECM of GRX cells, the peptide-targeted CS-NPs now show higher association than unmodified CS-NPs with GRX cells. In line with this, HP-NPs showed the highest increase in uptake/association when comparing the experiments performed in the presence versus in the absence of collagenase. This indicates that the degradation of collagen enabled the HP-NPs to make their way to PDGFR-β on the cell surface and to achieve increased accumulation through receptor-mediated endocytosis [38]. These findings and conclusions are bolstered by the notion that NP association/uptake did not change in HEK293 cells upon collagenase pretreatment (Figure 3E). However, repeating the association experiments in the presence of excess PDGFR-β-binding peptide or with NPs modified with control off-target peptides would undoutfully offer further confirmation.

### 3.3. siRNA-Containing Chitosan Nanoparticles Reduce Profibrogenic Gene Expression

**Figure 4:**
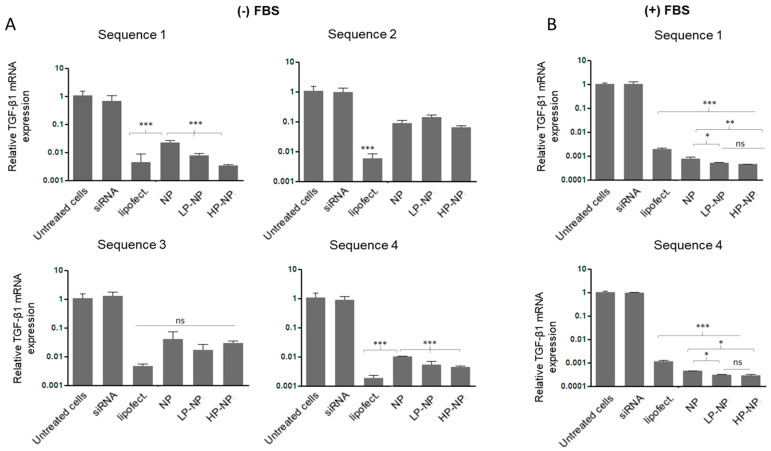
Transforming growth factor-beta (TGF-β1) gene silencing in GRX cells as determined by RT-qPCR (n = 3). Cells were treated with different sequences of TGF-β siRNA in free form (siRNA), transfected with Lipofectamine 2000 (lipofect.), encapsulated in unmodified NPs (NP), LP-NPs and HP-NPs in (**A**) serum-free cell culture medium and (**B**) medium supplemented with 10% fetal bovine serum (FBS). Statistical analysis was performed by GraphPad InStat software using a one-way analysis of variance test (ANOVA), where (*) *p* < 0.05, (**) *p* < 0.01 and (***) *p* < 0.001.

Four different anti-TGF-β1 siRNA were loaded into the NPs. Figure 4A shows TGF-β1 siRNA gene silencing results when cells were treated with siRNA in serum-free cell culture medium. When anti-TGF-β1-siRNA-loaded NPs were used in GRX cells, all three NP formulations (i.e., CS-NPs, LP-NPs and HP-NPs) were able to successfully reduce TGF-β expression as compared to untreated cells and to cells treated with naked siRNA. However, when cells were treated with anti-TGF-β1 siRNA pre-complexed with Lipofectamine 2000, the chitosan NPs showed inferior performance in serum-free medium conditions. However, experiments conducted in the absence of serum are not very realistic [39], particularly when the intended route of administration is the intravenous route. We therefore also performed experiments in cell culture medium supplemented with 10% FBS. Importantly, in this situation, the NPs outperformed the transfection reagent (Figure 4B). This is because most standard transfection reagents, such a Lipofectamine, are designed to function in the absence of serum, because serum proteins interfere with the formation of nucleic acid-loaded transfection agents, and/or because of increased susceptibility to degradation by nucleases [40,41]. A closer look at the NPs performance under serum-containing conditions shows that peptide-modified NPs were more potent in reducing TGF-β1 expression than unmodified CS-NPs. This can be attributed to the enhanced ability of such NPs to accumulate intracellularly, as opposed to localizing in the vicinity of the cell. In this case, the therapeutic agent is a large macromolecular compound, which, when unassisted, is unable to cross the cellular membrane and localize in the cytoplasm where its intended action is required [42]. This is confirmed by the inability of naked siRNA to reduce TGF-β1 expression either in the presence or absence of serum supplementation. CS-NPs show lower intracellular accumulation and higher ECM association when compared to LP-NPs and HP-NPs. Hence, the amount of intracellular siRNA is lower in the case of CS-NPs and higher with LP-NPs and HP-NPs, explaining their higher ability to reduce TGF-β1 expression. These results complement the in-vitro NPs association results. It is noteworthy that scrambled siRNA pre-complexed with Lipofectamine 2000 failed to reduce TGF-β expression as compared to untreated cells (Figure S6), indicating that the results observed are specific to the TGF-β siRNA used and not just to the treatment of cells with foreign genetic material.

While the quantification of TGF-β by Western blot would have been complimentary to qPCR result reports herein, the Western blot quantification of secreted TGF-β in NP-treated cells was not conducted. Utilizing its cationic amino groups chitosan has been demonstrated to bind with anionic TGF-β forming polyelectrolyte complexes [43]. Tsai et al., demonstrated that TGF-β was no longer detectable by Western blot when cells were treated with chitosan [43]. The latter, however, would not be problematic with qPCR experiments where intracellular mRNA were quantified as opposed to the extracellular secreted protein. Notwithstanding the above, this might indicate an added benefit for the use of chitosan in liver fibrosis. In addition to the ability to deliver functional TGF-β siRNA, chitosan NPs might also be able to sequester TGF-β in the liver and hence make it less available for further activation and propagation of the activated hepatic stellate cell population imparting synergistic benefit. In such cases, the treatment of GRX cells with unloaded CS-NPs followed by a comparison of TGF-β mRNA expression level by qPCR and TGF-β by Western blot would be insightful.

### 3.4. In-Vivo Nanoparticle Biodistribution

**Figure 5:**
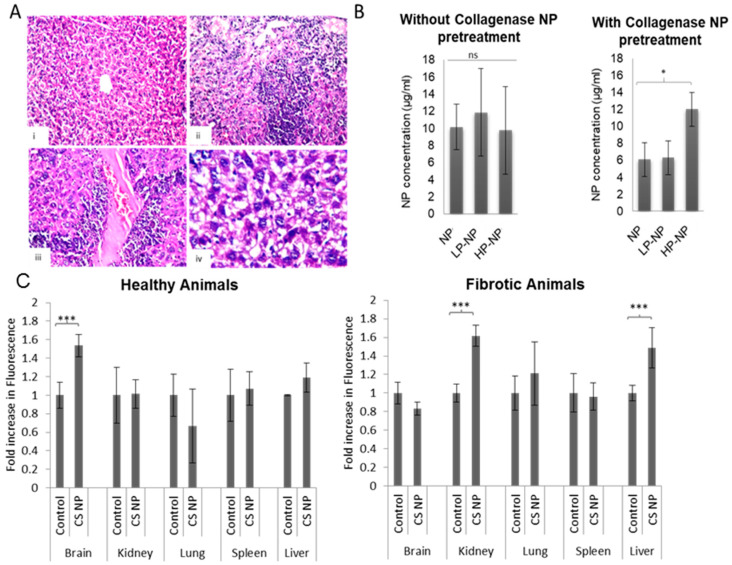
(**A**) Histopathological evaluation of liver sections obtained from healthy mice (i) and (ii-iv) mice receiving CCl_4_ (magnification 40×) (i) Normal liver architecture and intact cells obtained from healthy mice receiving olive oil. (ii) Focal necrosis with inflammatory cells aggregation in hepatic parenchyma. (iii) Massive inflammatory cell infiltration surrounding the dilated, congested central vein. (iv) Diffuse degenerated hepatocytes in the parenchyma. (**B**) NP accumulation in fibrotic livers as a function of platelet-derived growth factor receptor-beta (PDGFR-β) binding peptide density and collagenase-loaded NP pretreatment. (**C**) NP biodistribution in healthy and fibrotic animals. The results are expressed as the mean fold increase in fluorescence relative to untreated controls. Statistical analysis was performed by GraphPad InStat software using a one-way analysis of variance test (ANOVA), where (*) *p* < 0.05 and (***) *p* < 0.001.

Liver fibrosis was induced in mice using CCl_4_ to determine the ability of the NPs to accumulate at pathological sites following intravenous administration. Figure 5A (i) shows the normal histological morphology of the central vein and surrounding hepatocytes in the parenchyma of non-fibrotic livers in healthy animals. Liver sections of mice receiving CCl_4_ for 1 month showed focal necrosis with the aggregation of inflammatory cells in hepatic parenchyma, massive inflammatory cells surrounding dilated and/or congested central veins and diffuse degenerative changes in the hepatocytes in the parenchyma (Figure 5A, ii-iv), which are all hallmarks of liver fibrosis. Figure S5 shows the NP calibration curves obtained for CS-NPs, LP-NPs and HP-NPs in liver homogenates. In healthy mice that have received IV NPs, NPs were not detected in all healthy liver homogenates, at least not in a quantifiable manner using the constructed calibration curves (Figure S5). However, CS-NPs, LP-NPs and HP-NPs were all found to accumulate in fibrotic livers, indicating the ability of NPs to accumulate in capillarized fibrotic livers, assumingly through binding to the collagen-rich ECM. The lower liver accumulation observed for CS-NPs in non-fibrotic livers exemplifies the added value of chitosan-mediated collagen binding for successful disease targeting. This is of particular interest since most intravenously administered NPs tend to strongly accumulate in the liver where they are sequestered by Kupffer cells [31,44,45]. The extent of Kupffer cell accumulation is, however, a function of NPs’ physicochemical properties, with smaller and more hydrophilic NPs showing less Kupffer cell accumulation than larger hydrophobic ones [31,45]. For this reason, small-sized and relative hydrophilic CS-NPs showed low liver accumulation in healthy animals and significant liver accumulation was only observed in the presence of a high collagen density in fibrotic livers.

Figure 5B shows that, in fibrotic animals that did not receive collagenase-NPs to decrease collagen in the ECM, there was no difference in the extent of liver accumulation for the three NP formulations. Conversely, however, when animals were pre-treated with collagenase-NPs, the HP-NPs exhibited significantly higher accumulation in fibrotic livers as compared to unmodified NPs and LP-NPs. Interestingly, for both CS-NPs and LP-NPs, the extent of liver accumulation was reduced upon collagenase-NP pretreatment (approx. 1.7- and 1.9-fold respectively); this indicates that the digestion of ECM collagen reduces the NP accumulation, indicating that these NPs localize in the ECM as opposed to the aHSCs or even the Kupffer cells. Conversely, HP-NP accumulation increased upon collagenase-NP pretreatment, and, in cases such as those observed in the in-vitro association experiments, it could be concluded that these NPs do not accumulate in the liver as function of ECM matrix interaction or Kupffer cell sequestration but based on their interaction with the aHSCs. These conclusions are underpinned by the results of the in-vitro experiments where CS-NPs and LP-NPs did not show enhanced association in GRX cells upon collagenase pretreatment but HP-NPs showed a significant increase in association/uptake. This is in addition to the higher ability of HP-NPs to reduce TGF-β1 expression in GRX cells in comparison to unmodified and LP-NPs (Figure 4A). This is also confirmed by the results depicted in Figure 5C, which clearly indicate the change in CS-NPs biodistribution in fibrotic animals relative to healthy ones. In healthy animals, CS-NPs surprisingly accumulate in the brain. This is observed by the increase in fluorescence intensities obtained from CS-NPs receiving animals when compared to controls that have only received PBS. The accumulation of chitosan nanoparticles in the brain following IV administration has been reported previously [46,47]. In animals receiving IP CCl_4_, CS-NPs accumulate in kidneys and liver but not in the brain. Given that IP CCl_4_ injections have been reported to also result in kidney fibrosis [48,49] which is also characterized by excessive collagen deposition [50,51], it could therefore be implied that CS-NPs accumulate in organs of high collagen content. More importantly, based on the low NP accumulation in the spleen, which is also a major RES organ [52,53,54] and in the livers of healthy animals, the liver accumulation observed in fibrotic animals could not be mainly attributed to RES sequestration. The latter is underpinned by reports demonstrating that chitosan coating of NPs reduced their opsonization and phagocytosis [55,56]. Here, CS-NPs are also believed to accumulate as a function of chitosan–collagen interaction, and this further highlights the role of chitosan-collagen interaction in targeting of fibrotic diseases.

TGF-β1 is a pleiotropic polypeptide involved in the regulation of multiple processes, including adult stem cell differentiation, embryonic development, immune regulation, and inflammation, among others [57,58]. Accordingly, alterations in the TGF-β-signaling pathway contribute to a broad range of pathologies and hence TGF-β1 has been the therapeutic target of several diseases [8,57]. Generally, in addition to the use of siRNA, other means of reducing TGF-β levels have been reported. The blocking of TGF-β1 by means of monoclonal antibodies is one possible approach, besides other pharmacological strategies to inhibit the activation of TGF-β receptors, e.g., via the use of aptamers to attenuate downstream signaling [11,59]. All of these strategies, however, can result in the general (whole-body) inhibition of this factor, which may induce severe adverse effects [60,61]. Hence, drug targeting to the pathological site is explored to reduce toxicity and improve efficacy. In the case of liver fibrosis, one key strategy is delivery of therapeutic agents to the aHSCs, as performed in our study using anti-PDGFR-β peptide-targeted chitosan NPs. Given the double-edged role of collagen in the success of drug targeting by chitosan nanoparticles, combining anti-TGF-β1 siRNA-loaded HP-NPs with collagenase-containing NPs appears to be providing a viable strategy for further evaluation. The collagenase-NPs enable more efficient access to and delivery of siRNA into aHSCs, and at the same time they can help to degrade the collagen-rich scar tissue and thereby potentially assist in the resolution of fibrosis [25].

## 4. Conclusions

Collagen-binding chitosan nanoaprticles offer possible means for the targting of fibrotic livers. However, collagen-bound particles show limited ability in the interaction with target cells, offering limited intracellular delivery of therapeutic molecules, particlaury large macromolecules, such as siRNA. The modifcation of chitosan nanoparticles with targeting ligands, coupled with collagenase treatment, allows for increased NP uptake by target cells, with the intracellular delivery of therapeutics possibly imporving therapeutic outcomes.

## Supplementary Materials

The following are available online at https://www.mdpi.com/1999-4923/12/6/590/s1, Figure S1: Schematic representation of the in-vivo study. Figure S2: CS-NPs modification with increasing density of PDGFR-β targeting peptides, Figure S3: PDGFR-β expression by Western blot and RT-qPCR in GRX, LX2 and HEK293 cells. Figure S4: A comparison between the HD and ZP of unloaded CS-NPs and CS-NPs loaded with a model oligonucleotide, Figure S5: Collagenase cytotoxicity studies on GRX and HEK293 cells. Figure S6: Relative reduction in TGF-β mRNA expression determined by qPCR in GRX cells treated with scramble siRNA pre-complexed with Lipofectamin 2000, in serum free and serum supplemented cell culture medium, Figure S7: NPs calibration curves in fibrotic and healthy liver homogenates, Figure S8: NPs biodistribution in healthy and fibrotic animals, Table S1: Primer Sequences, Table S2: Thermal Profile, Table S3: TGF-β1 FlexiTube siRNA set box-Qiagen Germany (Cat. no. 1027416).
