# Supplementary material for: Targeting Activated Hepatic Stellate Cells Using Collagen-Binding Chitosan Nanoparticles for siRNA Delivery to Fibrotic Livers
Source: Pharmaceutics. 2020 Jun 25;12(6):590. doi: 10.3390/pharmaceutics12060590 (PMC7356502; doi:10.3390/pharmaceutics12060590)
Supplement: Supplementary file 1 [file pharmaceutics-12-00590-s001.pdf]

pharmaceutics

Supplementary Materials: Targeting Activated Hepatic Stellate Cells Using Collagen-Binding Chitosan Nanoparticles for siRNA Delivery to Fibrotic Livers

Menna Azzam, Sara El Safy, Sarah A. Abdelgelil, Ralf Weiskirchen, Anastasia Asimakopoulou, Federica de Lorenzi, Twan Lammers, Samar Mansour and Salma Tammam

Supplementary Methods

Validation of the Choice of Cell Line

Three cell lines; immortalized murine HSCs (GRX), immortalized human HSCs (LX2) and human embryonic kidney cells (HEK293) were tested for their basal expression levels of PDGFR-β and TGF-β. The three cell lines GRX, LX2 and HEK293 were cultured in full Dulbecco's Modified Eagle Medium (DMEM) supplemented with 10% FBS, 1% penicillin/streptomycin, 1% sodium pyruvate and 2 mM L-glutamate and seeded in three different 6-well plates at a density of 450,000, 400,000 and 350,000 cells/well respectively for two days. Cells were cultured at 37 °C in a humidified atmosphere with 5% CO_2. Western blot and quantitative real-time polymerase chain reaction (RT-qPCR) were used for the detection of PDGFR-β and TGF-β in GRX, LX2 and HEK293 cell lines.

Western Blot Analysis

For Western blot, the preparation of cellular protein extracts and conditions used for Western blot analysis was described previously [1]. Equal loading of lanes was demonstrated by probing with an antibody specific for glyceraldehyde 3-phosphate dehydrogenase (GAPDH). Primary antibodies used were GAPDH (sc-32233) (Dilution 1:1000 µL in 2.5%TBST), PDGFR-β (sc-432) (Dilution 1:1000 µl in 2.5% TBST), TGF-β1 (sc-146) (Dilution 1:200 µL in 2.5%TBST), all obtained from Santa Cruz Biotech (Santa Cruz, CA, USA) and secondary antibody used were horseradish peroxidase-conjugated anti-rabbit or anti-mouse IgG (Dilution 1:5000 µl in 2.5% TBST), (ThermoFisher Scientific, Schwerte, Germany).

Quantitative Real Time PCR

For RT-qPCR, total RNA was isolated using PureLink™ RNA Mini Kit in combination with PureLink® DNase (ThermoFisher Scientific). The total RNA was reverse transcribed into complementary DNA (cDNA) using SuperScript™ II Reverse Transcriptase (ThermoFisher Scientific). Quantification of real time PCR products was performed with the SYBR Green method using SYBR™ Green PCR Master Mix (ThermoFisher Scientific). Table S1 lists the primers used and Table S2 shows details of the thermal profile used. All primers were designed with the Universal Probe Library System Assay Design using the accession number of the gene of interest. All primers are validated according to the MIQE guidelines to ensure efficiency and reproducibility.

Table S1. Primer sequences.

Mouse gene	Primer sequence (5′→ 3′)
GAPDH	for: 5′-ACTGCCACCCAGAAGACTG-3′
	rev: 5′-CACCACCCTGTTGCTGTAG-3′
PDGFR-β	for: 5′-TCAAGCTGCAGGTCAATGTC-3′
	rev: 5′-CCATTGGCAGGGTGACTC-3′

TGF-β1	for: 5′-TGGAGCAACATGTGGAACTC-3′
	rev: 5′-CAGCAGCCGGTTACCAAG-3′
α-SMA	for: 5′ -TGACAGAGGCACCACTGAACC -3′
	rev: 5′- TCCAGAGTCCAGCACAATACCAGT-3′
**Human gene**	**Primer sequence (5′→ 3′)**
GAPDH	for: 5′- AGCCACATCGCTCAGACAC-3′
	rev: 5′- GCCCAATACGACCAAATCC-3′
PDGFR-β	for: 5′- ACTACTACGCCAAGGAGGTCAC-3′
	rev: 5′-TGCTTGAACTTGTCATAGATTTCG-3′
TGF-β1	for: 5′- CATCTGCAAAACCACCATTG-3′
	rev: 5′-GAGACGTTGATGGATGACACC-3′
α-SMA	for: 5′-CCTATCCCCGGGACTAAGAC -3′
	rev: 5′- AGGCAGTGCTGTCCTCTTCT-3′

Table S2. Thermal profile.

Step	Temperature(°C)	Duration (sec)	Cycle
Initialization	50	120	Hold
Denaturation	95	15	40 cycles
Annealing	60	60	
Elongation	72	60	
Dissociation	95	60	Hold
	55	30	
	95	30	

The relative quantification was calculated according to the following equation:

ΔCt = Ct (gene test) − Ct (endogenous control; GAPDH)

ΔΔCt = ΔCt sample − ΔCt calibrator (α-SMA)

RQ = Relative quantification = $2^{-\Delta\Delta Ct}$, where the RQ is the fold change compared to the calibrator (untreated sample, normal group) [2].

Collagenase Cytotoxicity Studies on GRX and HEK 293 Cells

HEK293 and GRX cells were seeded in 96 well plates at a density of 30,000 cells/well and allowed to adhere overnight. Cells were then treated with increasing concentrations of collagenase solution (0.005, 0.01, 0.025, 0.05, 0.1, 0.25, 0.5, 1, 2, and 4 mg/ml) and incubated for 24 hours. Thereafter, collagenase-containing cell culture media were aspirated and cell viability relative to untreated control was determined by the MTT assay [3].

Table S3. TGF-β1 FlexiTube siRNA set box-Qiagen Germany (Cat. no. 1027416).

Seq.	Target sequence	Passenger strand	Guide Strand
1	CCGAAGCGGACTACTATGCTA	5′-GAAGCGGACUACUAUGCUA d(TT)-3′	3′-d (GG) CUUCGCCUGAUGAUACGAU-5′
2	CAGCAAGGTCCTTGCCCTCTA	5′-GCAAGGUCCUUGCCCUCUA d (TT)-3′	3′- d (GT) CGUUCCAGGAACGGGAGAU-5′
3	CAGTATATATATGTTCTTCAA	5′-GUAUAUAUAUGUUCUUCAA d(TT) 3′	3′-d (GT) CAUAUAUAUACAAGAAGUU-5′
4	CCGCAACAACGCCATCTATGA	5′-GCAACAACGCCAUCUAUGA d(TT)-3′	3′-d (GG) CGUUGUUGCGGUAGAUACU-5′

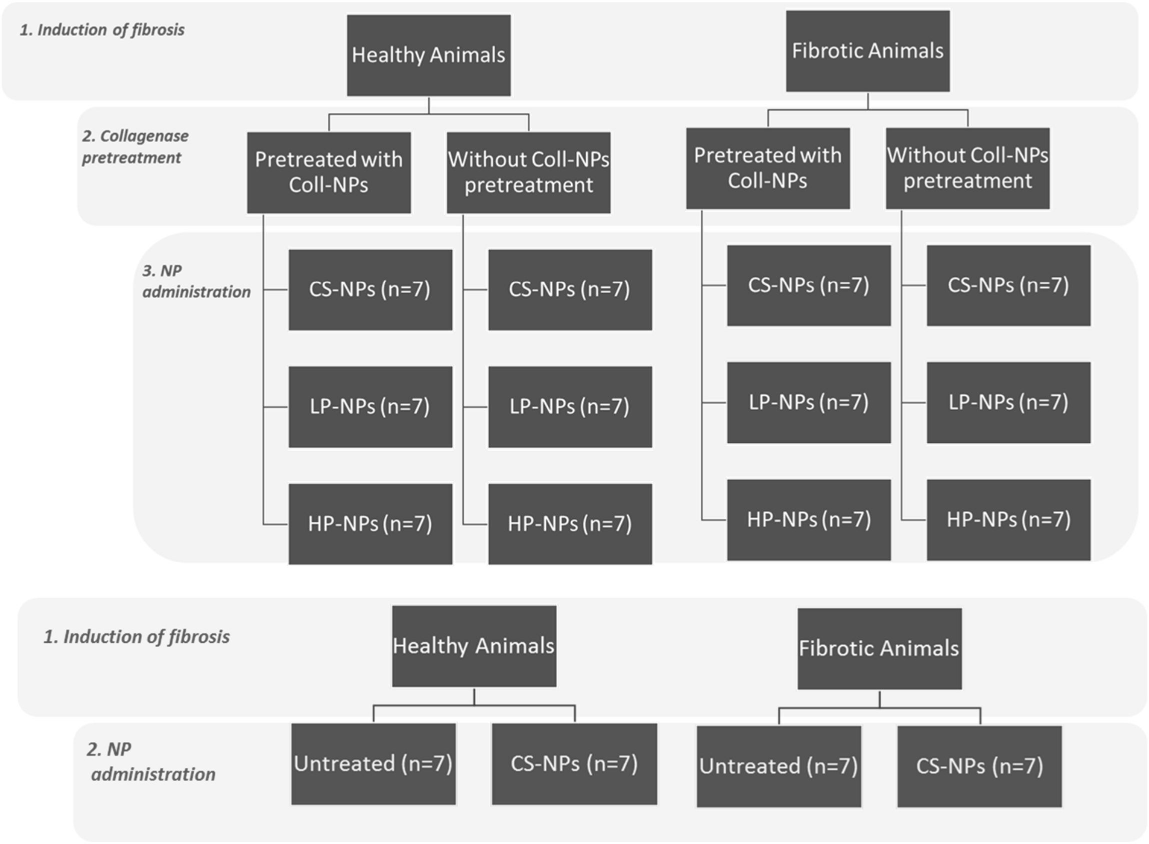

Figure S1. Schematic representation of the *in-vivo* study.

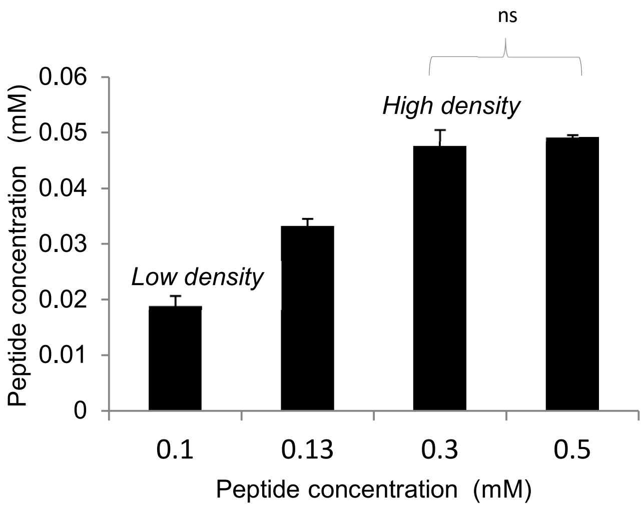

Figure S2. CS-NP modification with increasing density of PDGFR-β targeting peptides. Results are presented as mean ± SD of peptide concentration tagged on CS-NP, n = 3. When increasing concentrations of peptide were added to CS-NP, a higher density of peptide was tagged on to the NP surface, until the NP surface was saturated with peptide. At this point any further increase in the concertation of peptide added did not result in a change in peptide density on the surface of the NP. At saturation the NPs were believed to have a high peptide density and low peptide density was assigned accordingly.

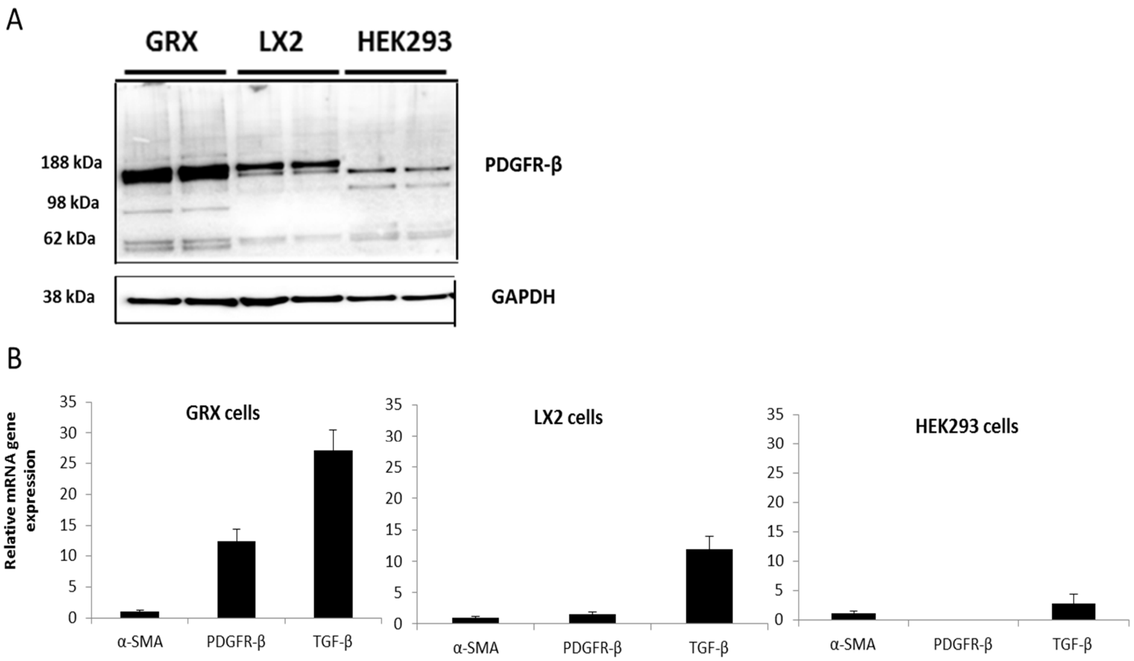

Figure S3. Western blot and RT-qPCR. (**A**) Western Blot results indicating the high expression of PDGFR-β in both GRX and LX2 and much lower extent in HEK293 cells. (**B**) Results of RT-qPCR showing that the relative PDGFR-β and TGF-β expression is higher in GRX and LX2 cells than in HEK293 cells when normalized to α-SMA content. Since GRX cells showed the highest expression of both PDGFR-β and TGF-β, it was therefore selected as cell line of choice for further experiments. HEK293 cells were selected as control cells given their low expression of both genes.

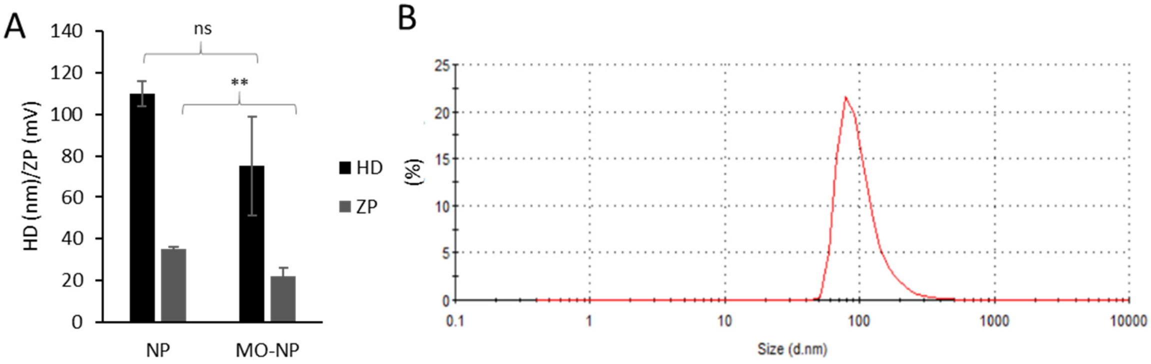

Figure S4. (**A**) A comparison between the HD and ZP of unloaded CS-NP and CS-NP loaded with a model oligonucleotide (MO), results are presented as mean ± SD for n = 3. (**B**) Hydrodynamic diameter distribution characterization by Dynamic Light Scattering of MO-NP. Statistical analysis was performed by GraphPad InStat software using unpaired t-test, where (*) $p < 0.05$ and (**) $p < 0.01$.

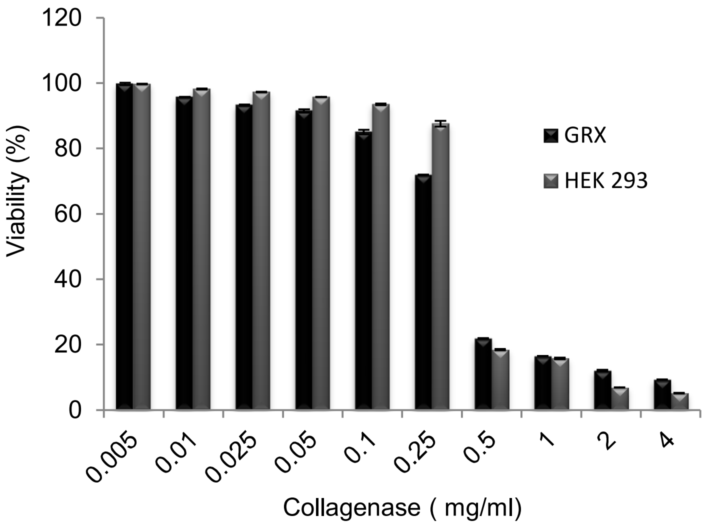

Figure S5. Collagenase cytotoxicity studies on GRX and HEK293 cells. Results are expressed as mean viability % ±SD, n=4 obtained for GRX and HEK 293 cells when treated with increasing concentrations of collagenase for 24 hours. Collagenase did not show significant cytotoxicity towards both cells when treated with concentrations up to 0.25 mg/ml. For this reason, the NP uptake experiments in GRX and HEK 293 cells were repeated after cells were pretreated with 0.2 mg/ml collagenase.

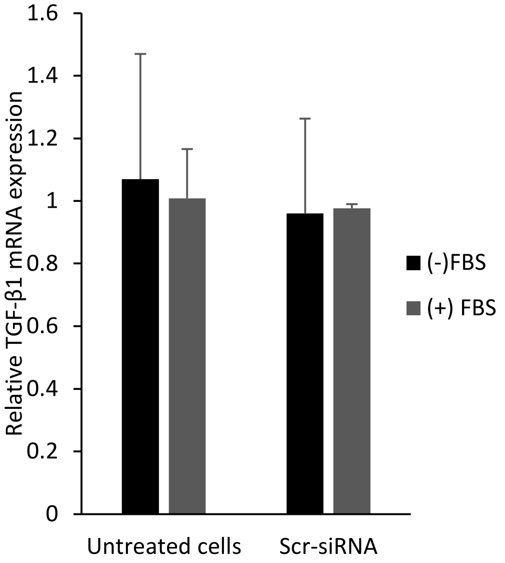

Figure S6. Relative reduction in TGF-β mRNA expression determined by qPCR in GRX cells treated with scramble siRNA pre-complexed with Lipofectamine 2000, in serum free and serum supplemented cell culture medium for n = 3.

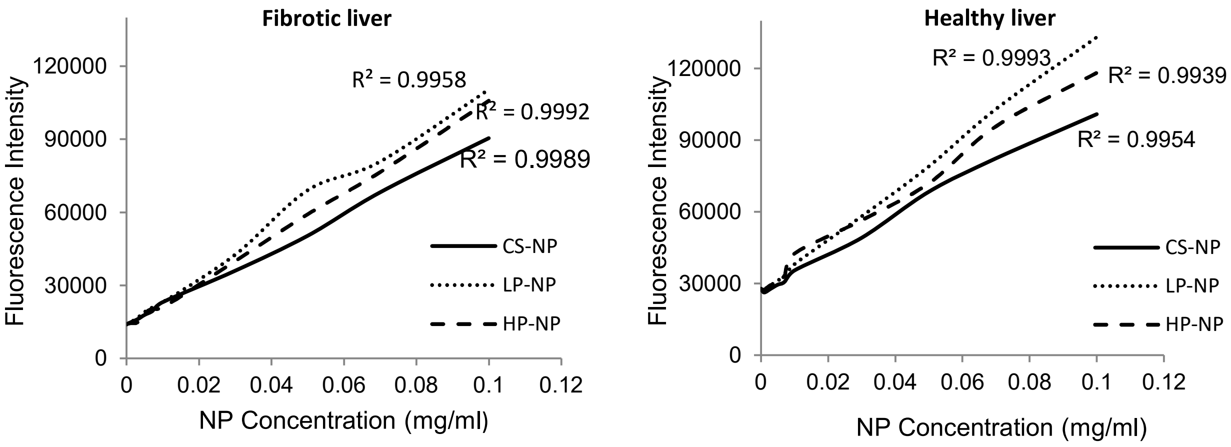

Figure S7. NP calibration curves in fibrotic and healthy liver homogenates.

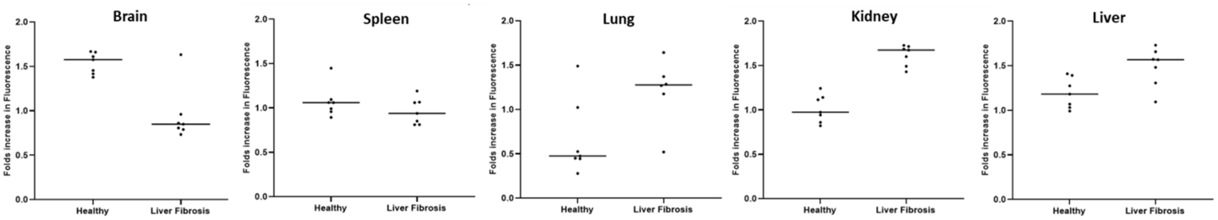

Figure S8. NP biodistribution in healthy and fibrotic animals.

References

1. Borkham-Kamphorst E, Steffen BT, Van de Leur E, Tihaa L, Haas U, Woitok MM et al. Adenoviral CCN gene transfers induce in vitro and in vivo endoplasmic reticulum stress and unfolded protein response. Biochimica et Biophysica Acta (BBA)-Molecular Cell Research. 2016;1863(11):2604–12.
2. K. J. Livak, Schmittgen TD. Analysis of Relative Gene Expression Data Using Real-Time Quantitative PCR and the 2−ΔΔCT Method. Methods 2001;25(4):402–8.
3. Buch K, Peters T, Nawroth T, Sänger M, Schmidberger H, Langguth P. Determination of cell survival after irradiation via clonogenic assay versus multiple MTT Assay—A comparative study. Radiation Oncology 2012;7(1):1. doi:10.1186/1748-717X-7-1.
